# Debundling of SWCNTs Using a Non-Toxic, Low Carbon Footprint Dispersant

**DOI:** 10.3390/polym17223007

**Published:** 2025-11-12

**Authors:** Carlos Salas-Bringas, Maria Gastony

**Affiliations:** 1 Borregaard ASA, Hjalmar Wessels vei 6, 1701 Sarpsborg, Norway; 2 Borregaard USA, 100 Grand Avenue, Rothschild, WI 54474, USA; maria.gastony@borregaard.com

**Keywords:** debundling, lignosulfonate dispersant, single-walled carbon nanotubes (SWCNTs), rheology

## Abstract

A fully aqueous, N-methyl-2-pyrrolidone–free strategy for debundling single-walled carbon nanotubes (SWCNTs) is reported using the renewable dispersant Vanisperse^®^ LI. Dispersions at 2 mg mL^−1^ were subjected to probe ultrasonication at 0.3 W mL^−1^ and evaluated using oscillatory rheology. Complex viscosity (η*) exhibited a transient maximum (~75 min) consistent with the formation of a percolated fibrous network, followed by a decline as debundling progressed. An optimum dispersant coverage of ~1.5 mg m^−2^ minimized η*, while overdosing likely induced multilayer adsorption and bridging seen by a rapid increase in η*. A two-stage centrifugation at 10,000× *g* yielded storage-stable suspensions of debundled SWCNTs without ultracentrifugation. SEM confirmed substantial debundling into thin fiber-like bundles. By formulating a dispersion with a dispersant that has a significantly lower cradle-to-gate carbon footprint than both fossil-based and bio-based alternatives such as CMC, this work presents a more sustainable approach to producing debundled SWCNT dispersions for advanced material applications.

## 1. Introduction

Lignosulfonates are sulfonated, water-soluble lignin polymers obtained from the sulfite pulping process. They behave as amphiphilic polyelectrolytes with strong surface activity, metal complexing ability, and colloidal stabilization. These properties underpin a broad application portfolio across industries, including cement and concrete [1,2], dyes and pigments [3], oil and gas [4], UV protection [5], water treatment [6], etc.

Single-walled carbon nanotubes (SWCNTs) are one dimensional sp^2^ carbon nanomaterials with very high aspect ratio, high electrical and thermal conductivity, and exceptional mechanical resilience. In lithium-ion electrodes, SWCNTs are widely explored as conductive additives and percolating networks that lower the electronic percolation threshold, homogenize current distribution, and mechanically bridge active particles [7,8]. This function is particularly valuable for silicon containing anodes that undergo large expansion, where SWCNT networks can mitigate pulverization and improve cycling [9]. Recent studies have shown that SWCNTs promote more uniform lithiation and volume change accommodation and demonstrate strong performance even at sub percent loadings [10,11]. Beyond their use as additives, SWCNT mats and films are also investigated as binder free or freestanding electrodes and as lightweight current collector replacements, though these approaches are still evolving toward manufacturability [12].

Water-based processing of lithium-ion battery anodes, for example, graphite or silicon graphite formulated with CMC or SBR binders, is now a common industrial practice. This approach eliminates the need for N-methyl-2-pyrrolidone (NMP) solvents, reduces costs and environmental as well as social burdens, and simplifies both drying and solvent recovery [13]. In line with this transition, recent research and industrial practices are increasingly adopting water-soluble battery additives such as CMC, SBR, PVP, and PAA. At a less explored level, some lignosulfonates are also gaining attention.

A fundamental obstacle to utilizing SWCNTs is that, as produced, they occur in bundles held together by van der Waals forces. Debundling is therefore essential to fully realize their intrinsic properties. The debundling challenge arises because aqueous media have high surface tension and SWCNT surfaces are hydrophobic. Without appropriate dispersants and energy input, such as shear or sonication, re-aggregation is rapid. Reported strategies include the use of surfactants, polymer wrapping, and organic solvents. Although NMP has been widely used, it is classified as a reproductive toxicant under the European CLP Regulation (EC No 1272/2008). Contemporary reviews of lithium-ion electrode processing therefore highlight a shift to aqueous binders, especially for anodes, to avoid health, environmental, and energy burdens [14].

From a sustainability perspective, the estimated cradle-to-gate carbon footprints of commonly used fossil-based dispersants for SWCNT applications in the battery industry are approximately 7–8 kg CO_2_e·kg^−1^ for polyvinylpyrrolidone (PVP) [15] and about 6–8 kg CO_2_e·kg^−1^ for sodium polyacrylate (PAA) [16], while poly(vinyl alcohol) (PVOH) shows site-specific values around 2.47 kg CO_2_e·kg^−1^ (Frankfurt, Germany) [17]. Bio-based alternatives, such as cellulose derivatives including carboxymethyl cellulose (CMC), exhibit similar or slightly higher values, typically in the range of 3–5 kg CO_2_e·kg^−1^ (approximate range inferred from comparative analysis) [18]. In contrast, some lignosulfonates, such as Vanisperse^®^ LI, report significantly lower footprints, around 0.315 kg CO_2_e·kg^−1^ (site-specific, Sarpsborg, Norway) [19]. Although these carbon footprint estimates are derived from LCAs using varying calculation methods, and minor discrepancies among methods are expected, the consistently lower value reported for Vanisperse^®^ LI underscores its potential as a low-impact dispersant.

Taken together, the need to effectively debundle SWCNTs in aqueous media using polymeric or bio-based dispersants, combined with the industry-wide shift toward water-based electrode processing to replace NMP, motivates the evaluation of the especially engineered lignosulfonate Vanisperse^®^ LI as a sustainable dispersant for carbonaceous anodes. This communication outlines initial performance metrics and processing parameters, emphasizing key variables such as SWCNT concentration, dispersant concentration, and sonication history, and aims to present preliminary findings that encourage further independent assessment of this alternative.

## 2. Materials and Methods

### 2.1. Materials

Single-walled carbon nanotubes (SWCNTs; Tuball^TM^) were obtained from the manufacturer OCSiAl S.A. (Luxembourg) and used as received. The as-supplied material exhibited a specific surface area of 922 m^2^g^−1^, an ash content of 2.09%, and a moisture content of 2.64%. The powder resistivity was 476 μΩ m, and the pH of an aqueous dispersion was 7.16. Particle size distribution, reported by the producer after 1 and 30 min of aqueous dispersion, showed D_50_ values of 283 μm and 146 μm, respectively. Metal impurities included Fe (1900 ppm), Co (9.7 ppm), and Ni (2.2 ppm).

### 2.2. Dispersant and Medium

Vanisperse^®^ LI (lignosulfonate-based dispersant, Borregaard ASA, Sarpsborg, Norway) and deionized water were used for all preparations.

### 2.3. Preparation of SWCNT Dispersions

SWCNT dispersions were prepared at a concentration of 2 mg mL^−1^ in deionized water. Vanisperse^®^ LI was added at varying dosages expressed as surface coverage (0.5–3.0 mg m^−2^) to evaluate its effect on dispersion quality.

### 2.4. Sonication Procedure

Dispersions were sonicated using a QSonica ultrasonic probe (Qsonica LLC, Newtown, CT, USA). The sonicator was operated at an amplitude adjusted to deliver a power density of approximately 0.3 W mL^−1^, as reported by Yang et al. [20], corresponding to an oscillating power output of 29–30 W. Sonication was performed for 1 h in pulse mode (10 s on, 10 s off) to prevent overheating. The sample container (100 mL beaker) was immersed in an ice-water bath to ensure low temperature during processing.

### 2.5. Debundling Process

Pre-screening for SWCNT concentration. An initial visual screening was performed to determine a suitable SWCNT concentration for ultrasonic dispersion. Dispersions were prepared at different concentrations in deionized water and visually assessed for viscosity and processability. At higher loadings (e.g., 8 mg mL^−1^), the dispersion rapidly thickened into a gel-like slurry that dampened ultrasonic propagation, as seen by the absence of surface motion during treatment. In contrast, at ~2 mg mL^−1^, visible surface disturbance indicated good ultrasonic propagation, while maintaining a solids content of interest for practical yield. This concentration was therefore selected as a practically relevant compromise between dispersion quality and solids loading rather than an absolute optimum for sonication efficiency, and it falls within the concentration range (1–8 mg mL^−1^) previously employed by Yang et al. [20] for SWCNT dispersion. Two short videos provided in the Supplementary Information (Sonication of 8 mg mL^−1^ SWCNTs and Sonication of 2 mg mL^−1^ SWCNTs) illustrate these contrasting behaviors.

Optimization of dispersant dosage. Following the determination of suitable concentration and sonication conditions, the effect of Vanisperse^®^ LI dosage was evaluated. Dispersions were prepared with Vanisperse^®^ LI at surface coverages ranging from 0.5 to 3.0 mg m^−2^ relative to the SWCNT surface area. Each formulation underwent the same sonication protocol described above.

### 2.6. Suspension of Debundled SWCNTs

The separation protocol employed in this study was adapted from Yang et al. [20]. A two-step centrifugation approach was implemented following the ultrasonic dispersion of SWCNTs (2 mg mL^−1^) in aqueous media containing Vanisperse^®^ LI at an optimized surface coverage of 1.5 mg m^−2^. The initial sonication (0.3 W mL^−1^, 60 min) was followed by centrifugation at 10,000× *g* for 60 min to remove large aggregates, after which the supernatant underwent a secondary sonication and a final centrifugation at 10,000× *g* for 15 min. This protocol yielded a highly debundled SWCNT suspension without resorting to the ultracentrifugation used by Yang et al. [20]. The reduction in centrifugal force, enabled by the viscosity-lowering effect of Vanisperse^®^ LI, renders the process more representative of the conditions achievable in battery manufacturing environments, where high-throughput, cost-effective separation is critical.

### 2.7. Rheological Measurements

Rheological characterization was performed using a rotational rheometer (MCR 102e, Anton Paar GmbH, Graz, Austria) equipped with a plate–plate geometry (50 mm diameter). All measurements were carried out in oscillatory shear at an angular frequency of 10 rad s^−1^, the linear viscoelastic region (LVR) was captured by the strain sweeps (0.01–100%) and the temperature was kept to 293.15 K. This approach was chosen in preference to shear rate sweeps, since oscillatory measurements in the LVR minimize shear induced artifacts such as wall slip, elastic instabilities, and shear banding. The average complex viscosity (η*) from the initial plateau region was used as an indicator of dispersion quality.

### 2.8. Microscopy

SEM microscopy (NeoScope JCM-7000 Benchtop Scanning Electron Microscope, Jeol, Tokyo, Japan) and an optical microscope (Olympus BX51, Olympus Corporation, Tokyo, Japan) were employed to evaluate the dispersion state of SWCNTs during and at the end of the separation process. For SEM, the sample was imaged with the secondary electron detector under high vacuum with an accelerating voltage of 15 kV at a working distance of 12.1 mm. The software for the optical microscope was PreciV capture 1.2 (Olympus) and for SEM imaging and analysis were NeoScope version 2.000 (Jeol) and Smile View Lab V3.20.4, respectively.

## 3. Results and Discussions

### 3.1. As-Received SWCNTs and Implications for Processing

**Figure 1:**
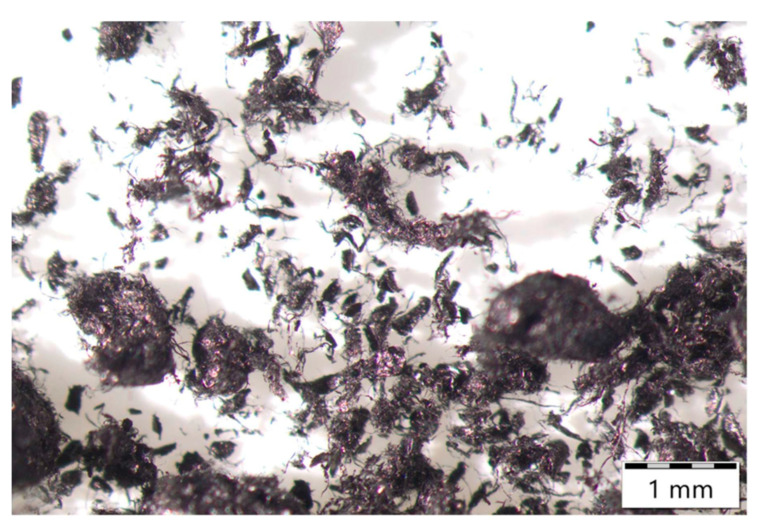
Dry SWCNT morphology.

The supplier reported high specific surface area with modest ash and moisture, and the presence of residual catalyst metals dominated by Fe with minor Co and Ni, which is consistent with typical as-produced SWCNT powders. Optical inspection (Figure 1) revealed multiscale agglomerates and rope-like bundles from tens of micrometers to the millimeter scale, in line with strong van der Waals interactions and entanglement characteristic of as-produced SWCNTs [20,21]. The coarse effective particle size disclosed by the supplier (D50 in the 10^2^ μm range) confirms that substantial debundling and colloidal stabilization are prerequisites for application relevant processing. Accordingly, Vanisperse^®^ LI and controlled energy input were employed to promote debundling and maintain dispersion quality.

### 3.2. Pre-Screening of Solids Content for Effective Ultrasonication

A pre-screening step established a workable SWCNT concentration range for sonication. At high loading (~8.06 mg mL^−1^), the dispersion rapidly thickened within less than one hour of probe sonication, forming a gel-like slurry that likely attenuated acoustic propagation and reduced cavitation efficiency. Microscopy of diluted aliquots showed large agglomerates alongside short and long fibrillar features. Guided by handling considerations and literature on viscosity limited ultrasonic debundling [22], a solids concentration of 2 mg mL^−1^ was selected for the systematic study.

### 3.3. Time-Dependent Debundling Under Controlled Sonication Power

**Figure 2:**
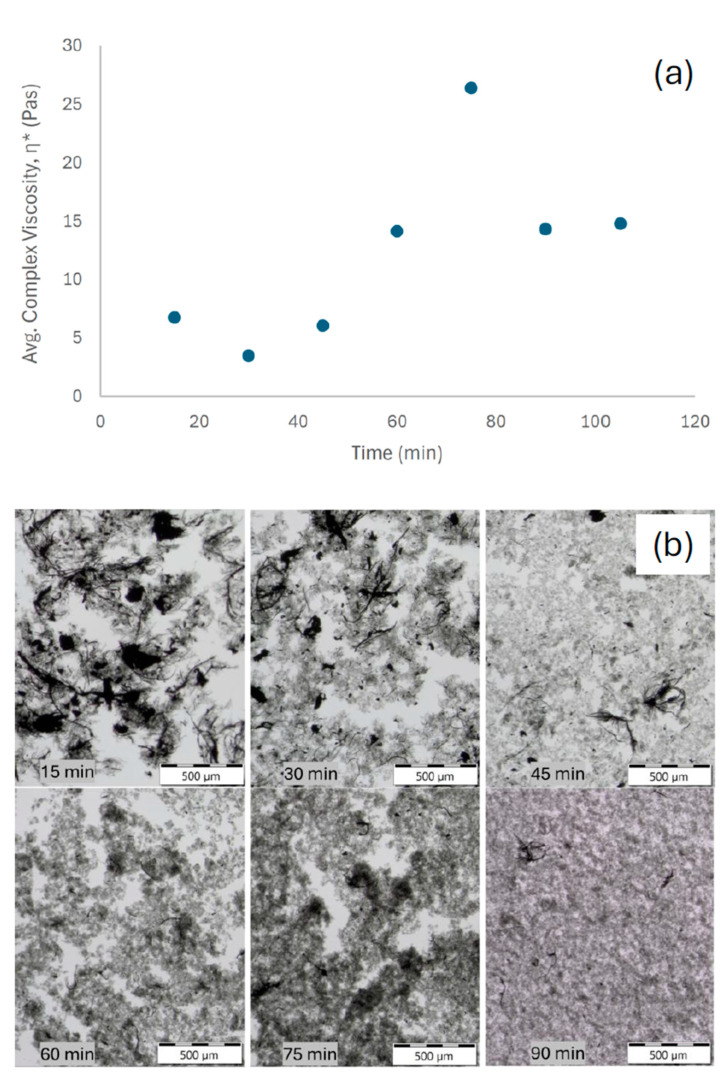
(**a**) Time-dependent complex viscosity of SWCNT dispersions (2 mg mL^−1^, 1 mg m^−2^ Vanisperse^®^ LI, 0.3 W mL^−1^). (**b**) Corresponding bright-field optical micrographs (15, 30, 45, 60, 75, and 90 min) showing the qualitative evolution from dense clusters to finer and shorter bundles.

Time resolved experiments of 1 mg m^−2^ Vanisperse^®^ LI and 0.3 W mL^−1^ (pulse mode, thermal control) suggest a non-monotonic trajectory for η* (Figure 2a), an initial decrease from the as-mixed state, a pronounced rise to a maximum near 75 min, and a subsequent decrease upon extended treatment. Qualitative optical micrographs acquired at matching time points (Figure 2b) show a transition from dense clusters to finer bundles, consistent with the viscosity evolution and dispersion kinetics reported by Yang et al. [20], who observed an analogous rise–decay behavior during SWCNT ultrasonication. This behavior is consistent with a transient, percolated network of partially debundled, long aspect-ratio filaments that enhances elastic resistance, followed by progressive disintegration and/or shortening that reduces interparticle connectivity and viscoelasticity [20]. Although extended sonication promotes fluidization, over-exposure can shorten nanotubes and compromise intrinsic properties, thus, time energy input must balance debundling with structural preservation [20,22].

#### Interpretation

In low-strain oscillatory rheology, η* is governed by the connectivity and arrangement of elements in the CNT network, providing a sensitive proxy for dispersion state while avoiding high shear artifacts (e.g., elastic instabilities, wall slip) that can distort steady flow curves. The observed maximum suggests an intermediate state in which Vanisperse^®^ LI has wetted and partially separated bundles, but hydrodynamic contact and physical entanglement remain sufficient to create a relatively strong viscoelastic network. Additional acoustic energy then suppresses connectivity as stabilization progresses.

### 3.4. Role of Vanisperse^®^ LI Dosage at Fixed Solids Loading

**Figure 3:**
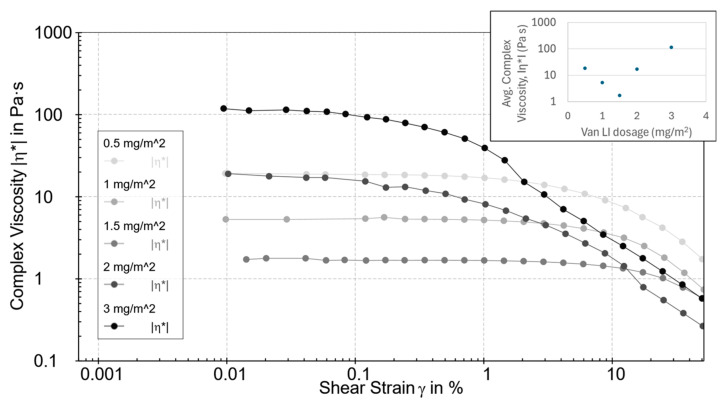
Complex viscosity (|η*|, mPa·s) as a function of shear strain (γ, %) for SWCNT dispersions with varying Vanisperse^®^ LI dosages (0.5–3.0 mg m^−2^). The inset shows the LVR-averaged |η*| versus dosage, indicating an optimum near 1.5 mg m^−2^.

**Figure 4:**
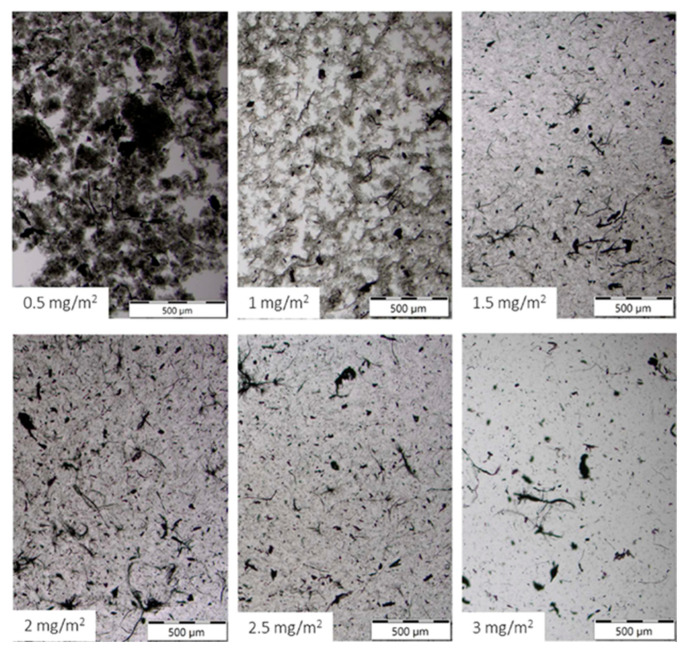
Optical micrographs across dosage series.

At 2 mg mL^−1^ SWCNTs, varying Vanisperse^®^ LI surface coverage from 0.5 to 3.0 mg m^−2^ uncovered a clear optimum near ~1.5 mg m^−2^, where the average η* (Figure 3) in the LVR reached a minimum (log scale spanning orders of magnitude). Below this level, incomplete surface coverage leaves attractive contacts and micro bundles. Above ~2 mg m^−2^, η* increased, and the liquid phase appeared slightly more clarified (Figure 4), consistent with bridging flocculation or multilayer adsorption of lignosulfonate at high concentration, as documented for lignin-derived polyelectrolytes [6,23].

**Figure 5:**
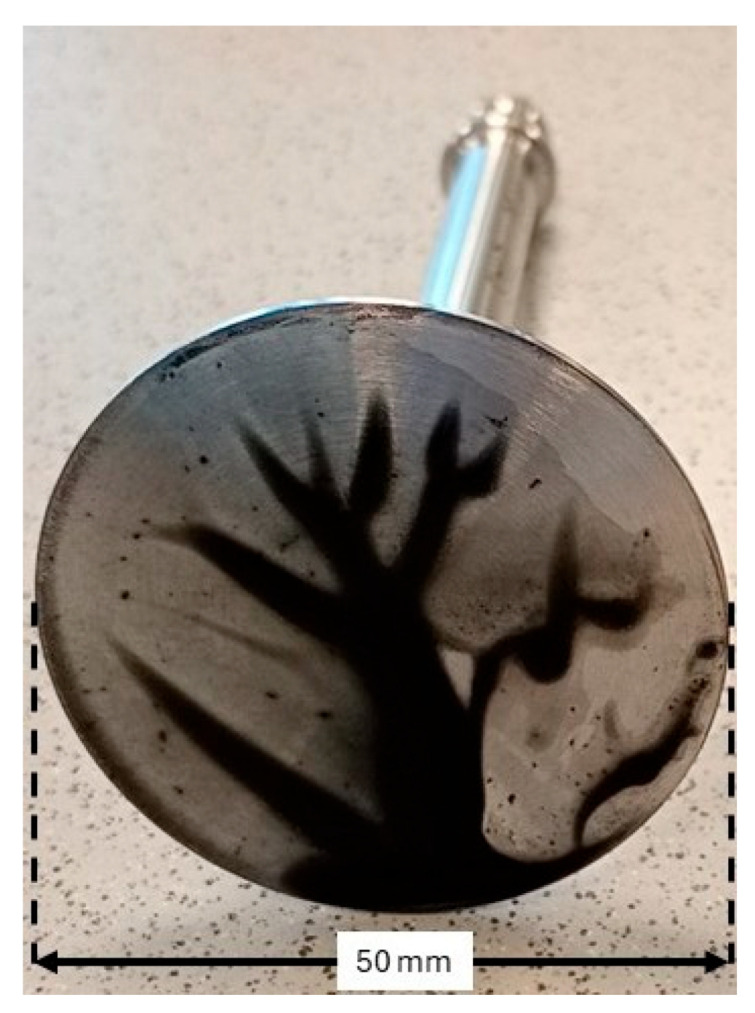
Residual slurry on the rheometer’s top plate after plate–plate measurements, showing visible black spots indicative of large SWCNT agglomerates.

Post-measurement deposits, visible as black spots on the rheometer top plate (Figure 5), were observed at high dosages (>2 mg m^−2^) but were absent at dosages around ~1.5 mg m^−2^. This supports the hypothesis that overdosing Vanisperse^®^ LI promotes the formation of large CNT aggregates through bridging flocculation.

### 3.5. Mechanistic Picture

Vanisperse^®^ LI provides stabilization through electrostatic repulsion and steric hindrance on graphitic surfaces. At sub-monolayer coverage, inter-bundle attractions remain significant. Near monolayer saturation, steric and electrostatic repulsive barriers are maximized, leading to optimal stabilization. At excess Vanisperse^®^ LI concentrations, self-association and multilayer adsorption can occur, enabling polymer bridging between coated SWCNTs and thereby re-increasing network connectivity and viscosity, as reported for other systems [6,23]. The dosage window that minimizes complex viscosity therefore defines a stability envelope for processing.

### 3.6. Two-Stage Separation Yields Debundled SWCNT Suspensions

**Figure 6:**
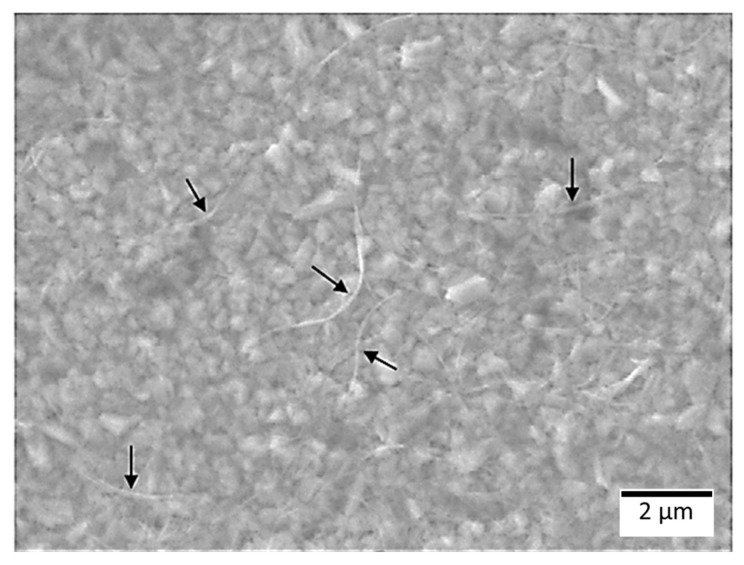
Scanning Electron Microscopy (SEM) image showing debundled SWCNTs deposited on a copper substrate. The arrows indicate thin, fiber-like bundles, representing partially debundled structures rather than fully individualized nanotubes, which cannot be resolved at this magnification. The granular background corresponds to the copper substrate.

A two-stage ultrasonication–centrifugation protocol, adapted from Yang et al. [20], was implemented under the identified best conditions (2 mg mL^−1^ SWCNTs, ~1.5 mg m^−2^ Vanisperse^®^ LI, 0.3 W mL^−1^). After the first sonication, centrifugation at 10,000× *g* for 60 min produced a supernatant (upper ~80%) and a pellet enriched in large bundles and impurities. Re-sonication of the supernatant, followed by a second centrifugation (10,000× *g*, 15 min), yielded a final supernatant comprising highly debundled SWCNTs, as confirmed by SEM imaging (Figure 6). The resulting fiber-like bundles exhibited an average thickness of 93 ± 15 nm, based on measurements from 13 individuals. Because these entities remained suspended under 10,000× *g*, the colloid is gravimetrically stable, any eventual sedimentation would most plausibly arise via slow flocculation rather than simple gravitational settling. However, no such behavior was observed after one month of storage.

### 3.7. Process Intensification and Green-Chemistry Perspective

Lignosulfonate dispersants can keep a low continuous phase viscosity at a given solids content which, through Stokesian scaling (*v* ∝ (Δ*ρd*2*g*)/(18*η*), where *v* is the settling velocity, Δ*ρ* the density difference between phases, *d* the particle diameter, *g* gravitational acceleration, and *η* the continuous-phase viscosity), can reduce required centrifugal field and/or residence time for an equivalent separation, thereby cutting energy and enabling higher throughput. These attributes, combined with the use of renewable chemistry and water as the processing medium, align with green-chemistry principles.

### 3.8. Colloidal Stability and Operating Window

Given the persistence of debundled SWCNTs in the supernatant at 10,000× *g*, sedimentation under quiescent gravity is not expected within practical timeframes. The dominant destabilization risk is bridging induced flocculation at overdosing of Vanisperse^®^ LI or through aging driven self-association, especially at elevated ionic strength.

### 3.9. Implications

Pairing a renewable dispersant with scalable ultrasonication and moderate-g centrifugation offers a practical route to debundle industrial SWCNT feedstocks. For applications such as battery conductive networks and coatings, the viscosity minimum at the optimized Vanisperse^®^ LI dosage facilitates high-solids processing and more uniform film formation, while the overdosing penalty underscores the need for precise formulation control.

### 3.10. Proposed Mechanistic Framework

The data support a sequential mechanism: (i) rapid adsorption of Vanisperse^®^ LI onto SWCNT sidewalls that mitigates inter-bundle van der Waals interactions; (ii) ultrasonication transforming hierarchical agglomerates into a percolated fibrous network, manifested as a viscosity maximum; and (iii) subsequent debundling at optimal coverage, where electrostatic repulsion and steric stabilization minimize interparticle connectivity, while higher dosages induce multilayer adsorption and bridging that reverse the viscosity trend.

### 3.11. Sustainability Spotlight

This study implements a renewable, water-based route for SWCNT debundling using Vanisperse^®^ LI, thereby avoiding N-methyl-2-pyrrolidone and other organic solvents. At the optimal dosage, Vanisperse^®^ LI lowers slurry viscosity, enabling reduced centrifugal field and/or residence time for equivalent separations. Vanisperse^®^ LI’s cradle-to-gate CO_2_ footprint is substantially lower than typical fossil-based dispersants (PVP, PAA, PVOH) and below bio-based alternatives such as CMC.

## 4. Conclusions

Vanisperse^®^ LI enables a fully aqueous, NMP-free route to debundle industrial SWCNT feedstocks under practical industrial conditions. At 2 mg mL^−1^ SWCNT and 0.3 W mL^−1^ sonication, an optimal Vanisperse^®^ LI coverage of ~1.5 mg m^−2^ minimized complex viscosity and disrupted the transient percolated network, whereas overdosing re-increased complex viscosity, consistent with multilayer adsorption and bridging. A two-step 10,000× *g* centrifugation produced storage stable (≥1 month) supernatants without the need for ultracentrifugation, with an ultrasonic energy demand of 0.3 kWh L^−1^. Coupling these processing benefits with the lower cradle-to-gate footprint of Vanisperse^®^ LI relative to common dispersants outlines an attractive, scalable pathway to lower impact SWCNT processing.

## Data Availability

The original contributions presented in this study are included in the article. Further inquiries can be directed to the corresponding authors.

## Supplementary Materials

The following supporting information can be downloaded at: https://www.mdpi.com/article/10.3390/polym17223007/s1, Video S1: Sonication of 2 mg mL^−1^; Video S2: Sonication of 8 mg mL^−1^.

## Abbreviations

The following abbreviations are used in this manuscript:

SWCNTs	Single-walled carbon nanotubes
NMP	N-methyl-2-pyrrolidone
CMC	Carboxymethyl cellulose
PVP	Polyvinylpyrrolidone
PAA	Poly(acrylic acid)/Sodium polyacrylate
PVOH	Poly(vinyl alcohol)
LVR	Linear viscoelastic region
SEM	Scanning electron microscopy/microscope
CO_2_e	Carbon dioxide equivalent
g	Relative centrifugal force (× g)
